# The King's Hospital Fund

**Published:** 1904-03-12

**Authors:** 


### HOSPITAL ADMINISTRATION.

### CONSTRUCTION AND ECONOMICS.

### THE KING'S HOSPITAL FUND.

#### A MESSAGE FROM THE KING. A GIFT OF NEARLY £5,000 A YEAR.

The annual meeting of the General Council of King Edward's Hospital Fund for London to receive the accounts and report of the General Council for the year 1903 was held last Tuesday morning at Marlborough House, the Prince of Wales being in the chair. Those present were the Bishop of London, the Chief Rabbi (the Rev. Dr. Adler), Viscount Duncannon, Lord Iveagh, Lord Farquhar, the Chairman of the London County Council (Lord Monkswell), the President of the Royal College of Physicians (Sir W. S. Church), Sir Savile Crossley, M.P., Sir Joseph Dimsdale, M.P., Mr. C. B. Stuart-Wortley, M.P., Sir Trevor Lawrence, Sir John Aird, M.P., Sir Henry Burdett, Sir W. J. Collins, Sir John Craggs, Mr. Sydney Buxton, M.P., Mr. F. M. Fry, Mr. Albert G. Sandeman, and Mr. H. C. Smith. Letters of regret were read from the following: the Duke of Bedford, the Bishop of Rochester, the Rev. J. Guinness Rogers, the Rev. T. Bowman Stephenson, Lord Rothschild, the President of the Hospital Sunday and Hospital Saturday Funds (the Lord Mayor), the Governor of the Bank of England (Mr. S. Hope Morley), the President of the Royal College of Surgeons (Mr. John Tweedy), Mr. Thomas Burt, M.P., Mr. William Latham, K.C., Mr. J. Pierpont Morgan, jun., Mr. Edgar Speyer, and Mr. Julius Wernher.

INCREASE IN SUBSCRIPTIONS.

In the absence of Lord Rothschild, Sir John Craggs presented the certified accounts of the Fund for the year ending December 31, 1903.

The Prince of Wales said: My Lords and Gentlemen,—I beg to move the adoption of the account of receipts and expenditure and the balance-sheet for the year ending December 31st, 1903.

Mr. Hugh C. Smith said: Your Royal Highness, my Lords, and Gentlemen,—We have seen from what has been said that, as compared with the year 1902, the annual subscriptions in 1903 were £4,100 more—the League of Mercy gave £2,000 more, and the income from investments was £16,200 more, owing to the munificent gifts of Lord Strathcona and Lord Mount Stephen, though, on the other hand, the donations were less. The amount taken from reserve this year so as to maintain the distribution to the hospitals on the same scale as in 1902 was £18,751 9s., but I should like to point out to your Royal Highness that in 1902 and 1903 the amount distributed to hospitals was exactly double what we distributed in 1901. I have great pleasure in seconding the resolution.

LEAGUE OF MERCY.

Sir Henry Burdett said: Your Royal Highness, my Lords and Gentlemen,—I rise to support the adoption of the accounts. As treasurer of the League of Mercy it is a great pleasure for me to point out that the growth of that League is far greater than it would appear by the figures in the contributions to the King's Fund this year as reported in these accounts. The League started, as you know, and I think it is well, in view of what was said after our last meeting, that it should be stated, to obtain small subscribers—subscribers of shillings and pence. I am glad to say that the £10,000 given by the League of Mercy to the Fund last year may be taken broadly as representing contributions of small sums, and it is very important indeed that you should understand that the people of London—the humbler people of London—are really taking a very practical interest in this Fund through the League of Mercy. Only the other day at a meeting where I was speaking there were over 1,000 people, and though no money was asked for the people pressed pence on the doorkeepers in passing out of the meeting. And I am glad to say that while I was putting on my coat in the entrance hall the doorkeepers brought in arms full of coppers. There is real value in these small contributions, and, as I know my colleague Sir William Collins can confirm, it brings the working classes intimately in touch with the League of Mercy, and by accepting personal service from them in the days of their health in the cause of the sick we gain the support and goodwill of the masses of the people of London. This must help us in furthering the good work and so enable us to collect the amount which we hope for, viz, £50,000 a year. I have very great pleasure in supporting the adoption of the accounts.

The motion was carried unanimously.

GENERAL COUNCIL'S REPORT.

Mr. H. C. Smith, chairman of the Executive Committee, read the report of the General Council for the year ended December 31st, 1903, which stated that the sums received during the year were, in addition to interest from investments: donations, £6,882 13s. 9d.; annual subscriptions, £32,834 1s. 3d.; from the League of Mercy, £10,000; and from the Shopping Day Committee a sum of £1,200 on account. The year 1902 was an exceptional one, and the council were accordingly in a position to recommend that the sum of £101,000 should be distributed among the London hospitals and convalescent homes. In the year just closed the amount received in donations showed a decrease, but the annual subscriptions had increased by £4,000, while the League of Mercy had contributed £2,000 more than in any previous year. During the five years of its existence the League has furnished in all £32,000 to the Fund. His Majesty the King, the patron, has been pleased to continue that deep interest in the Fund which he had taken from its inception; and the President, his Royal Highness the Prince of Wales, had given much time to the work of the Fund, and his continuous devotion to its welfare is evidenced by the speech he made on December 14th. Including the sum of £863 3s. 2d. received on account of the Coronation gift, but not included in last year's report, the receipts for the year 1903 amounted to £83,483 19s. After deliberate consideration the Council decided to recommend that £100,000 should be distributed among the hospitals and convalescent homes. This involved drawing on reserves to the amount of £18,751 9s. As the moneys thus drawn on were placed at the entire discretion of the Council this action was felt to be justified, having regard to present needs and in full reliance on continued sympathy and increased support from the charitable public. Every hospital to which a grant was made was inspected and reported on, and it was hoped that this work of the visitors might win, as it deserved, the confidence of the public in the administration of their contributions. The working expenses of the Fund during the year amounted to £2,218 17s., or less than $2\frac{3}{4}$ per cent. of the amount received, which sufficed to prove the economical manner in which the Fund was administered.

### The Prince's Speech.

The Prince of Wales said: My Lords and Gentlemen,—I have much pleasure in moving the adoption of the report for the past year, and on the whole I think we may be well satisfied with the results achieved. It could not be expected that the contributions to the Fund would be equal to those of the previous year, that of the Coronation. Moreover, I believe I am correct in stating that we have been, and are still, passing through a time of financial depression. Nevertheless, the accounts show the steady gradual progress of the Fund. Roughly speaking, the contributions are £30,000 more than in 1901, the last normal year. The sum distributed is £100,000—almost double that of 1901. (Hear, hear.) As I stated at our last meeting in December, I regret very much that it was necessary to draw on our reserves, and I trust that the ensuing year will enable us to provide £100,000 out of income without again having recourse to what may be considered an undesirable expedient. I shall be only echoing the unanimous opinion of the council in expressing our gratitude to all who, whether in large sums or small, have supported the Fund during the seventh year of its existence. As stated in the report, the investments of the Fund now standing in the names of Lord Rothschild and Mr. Hugh Colin Smith, taken at cost, comprise £234,037, while the securities received by the Fund, as valued at date of receipt, amount to £430,218, making a total of invested capital now owned by the Fund of £664,255. (Hear, hear.)

### Anonymous Gift of £4,668 a Year.

This position of affairs may be considered satisfactory. But we must endeavour to improve it. And I think you will all agree that we have already obtained very great encouragement when I tell you that within the last few days, to my surprise and gratification, I have received from a personal friend, who desires to remain anonymous, a letter which I should like to be allowed to read:—

"Sir,—I regret to learn that the assured income of the King's Hospital Fund from investments and other permanent sources only amounts to about £36,000 a year, which is £14,000 a year less than two years ago it was hoped the fixed income of the Fund from endowments, etc., would have reached by this time. It is clear to me that there is no better way of benefiting the sick poor of London than by supporting the King's Hospital Fund, and that it is a matter of the greatest importance to the London Hospitals generally, as well as to the success of the Fund itself, that it should as speedily as possible be placed in a position to enable it to make an annual distribution among the London hospitals of not less than £150,000.

"If the King's Fund had an assured income from investments and other permanent sources of, say, £50,000 a year, I believe that it would not be very long before the income from subscriptions and other sources would amount to £100,000 a year, making a total of £150,000 a year available for distribution, and placing the King's Fund in a position to exercise a most beneficial influence and control over the management of every London hospital. To help this most desirable object I have decided to anticipate a provision in my will, and, instead, to hand over to the King's Hospital Fund, by way of gift, securities representing the capital sum required to provide one-third of the £14,000 a year deficiency on condition that the remaining two-thirds—£9,332 per annum—is provided for. This offer to be good and to remain in force till 31st December next, 1904. I do not attach any other conditions to this offer, but would like to express a hope that your Royal Highness, as President of the King's Hospital Fund, may see your way to prevent any portion of the funds subscribed for the relief of the sick poor being diverted to purposes of medical education.

"Trusting your Royal Highness may be placed in a position to enable you to avail yourself of my offer." (Hear, hear.)

### A Personal Appeal.

You will, I know, share in my feelings of deep gratitude towards the writer of this letter for the substantial proof he gives of his interest and sympathy in the work of the Fund. I heartily agree with the ideal to which he hopes we should aspire. As a rule I am reluctant to make personal appeals. But having received this munificent offer I feel that it is my duty to invoke the support of those who are in a position to follow the example of this anonymous benefactor, and so enable us to secure the full results of his generous proposal. I should like to take this the first opportunity of personally expressing my warmest thanks to my kind friend for this noble effort to assist me in the work initiated by the King. (Hear, hear.) But while solicitous for the increase of our invested funds, I none the less attach the greatest importance to encouraging by every means at our disposal the gifts of small—of even the smallest sums. The total number of subscribers to the Fund is over 4,300. To this must be added the many subscribers to the League of Mercy. These statistics are satisfactory as showing that while we receive large donations the Fund is also becoming understood and I hope appreciated by the people. To bring this about was the original purpose of the Fund.

### The Generosity of the Dramatic Profession.

I take this opportunity of referring to one particular class of the community from which the London hospitals derive frequent, substantial, and unassuming help; that is, from a profession whose generosity and readiness to help those in adversity is almost proverbial—I mean the dramatic profession. (Hear, hear.) I sometimes think we do not fully realise how much of their valuable time and of their artistic powers are gratuitously given by the members of the stage for charitable purposes. (Hear, hear.) As usual the reports of the Visiting Committee have been of the greatest assistance in determining the annual distribution of the Fund. Many reforms of which the public hears nothing have been carried out on the recommendation of the Visiting Committee in addition to those enumerated and published in the report. Before closing these remarks I wish to offer my sincere thanks to the General Council, the honorary treasurer, the honorary secretaries, the members of the Visiting and Distribution Committees, and other honorary officials for their valuable services. It is through their zealous and self-sacrificing efforts that the work of the Fund is so efficiently and economically performed. I hope that they will be kind enough to again serve in their respective offices for another year. I have now great pleasure in moving the adoption of the report. (Hear, hear.)

The Working Classes and the Hospitals.

Sir John Aird said: Your Royal Highness, my Lords and Gentlemen,—I am very proud to have the opportunity of seconding the report which has been read by yourself, Sir, and I am sure it is the opinion of everybody present that it is carefully drawn up. I beg to second the adoption of the report.

The Bishop of London said: Your Royal Highness, my Lords and Gentlemen,—I am glad to support the adoption of the report, and I beg to congratulate your Royal Highness in the name of the whole council on the letter which you have read from your friend. In looking at the report there are one or two points I should like to remark on. First, the increase in the annual subscriptions. This is a matter of congratulation, for it is one of the most essential things for an undertaking like this. Then what Sir Henry Burdett has said on the matter of the help of the poor is very encouraging. In the old days I can recollect as much as £60 being collected round one stand in Victoria Park, showing how ready the working classes are to give their pence to the hospitals. Again, the number of beds which have been opened through the instrumentality of the Fund, no less than 443, is very encouraging. The Visiting Committee, I am perfectly sure by their excellent work, are keeping up the standard of hospital management in the whole of London. As I notice it is stated in the report that hospitals should be removed to larger and more densely-populated areas, on this question I would invoke the sympathy of the whole Council in the removal of King's College Hospital to the South of London, where I am sure it will meet a long-felt need. I have much pleasure in supporting the adoption of the report.

The report, on the motion of the Prince of Wales, was received and adopted.

Gratitude to the Prince.

Lord Monkswell said: May it please your Royal Highness, my Lords, and Gentlemen, the privilege has been accorded me of moving a vote of thanks to your Royal Highness for presiding over us on this occasion. As treasurer of a hospital in need of funds I have a special reason in congratulating your Royal Highness on the success of the Fund. I have the pleasure to move a vote of thanks to your Royal Highness for presiding here to-day. (Hear, hear.)

The Rev. Dr. Adler (the Chief Rabbi) said: I regard it as a high privilege to be permitted to second this vote of thanks. We all know how deep is the debt of gratitude which we owe to your Royal Highness. At all times the work of this Fund is valuable, and it is especially so at this period in the history of the London hospitals, when there are urgent schemes for the rebuilding and removal of certain of these institutions, and when there is so much financial depression existing, to which you, sir, have alluded. We therefore feel that the work of your Royal Highness is particularly valuable at the present time, and we fully realise how the success of this Fund is mainly, if not exclusively, due to the energy and sagacity with which you so ably preside over this Fund. We beg heartily to congratulate you, sir, on the success which has attended your efforts. We rejoice with you in the good news you have communicated to us to-day, and we feel that the best mode in which we can evidence our congratulations to you, sir, will be to use our best endeavours that the offer made may become an accomplished fact, and by working very hard to induce as large a number as possible of the entire community to contribute to this Fund. I beg leave to second this vote of thanks to your Royal Highness.

Message from King Edward VII.

The Prince of Wales said: I beg to thank you, my Lords and Gentlemen, for the very kind way in which you have received this vote of thanks to myself. As I have said before, I shall ever continue to take the greatest possible interest in the Fund, and I also rely on your very kind and able support, which you have always given me. I have a message from the King, which is for me to tell you that the interest he takes in this Fund is unabated, and is just the same as when he started the Fund, and he wishes me to congratulate the council and all concerned on the very efficient way in which it is administered.

The proceedings then terminated.

